# The History of Anæsthesia

**Published:** 1896-03

**Authors:** 


### Editorial.

### THE HISTORY OF ANÆSTHESIA.

We have received a pamphlet entitled "History of Anæsthesia or Painless Surgery," by Wm. R. Hayden, Bedford Springs, Mass.

The opening lines of the preface set forth the purpose of this brochure: "The following pages were written with an honest and unselfish desire to do justice to one whose inestimable services to humanity were persistently overshadowed by the most extraordinary perversion of facts—a fierce and unmanly attempt to prove that wrong was right and right was wrong."

The "one" referred to is Dr. W. T. G. Morton whose claims to the discovery of painless surgery, Dr. Hayden seeks to verify.

While this is the avowed purpose of the author, yet the claims of Long, Wells, and Jackson, are fully considered.

It is a pity that the history of this discovery, probably the most tremendous in the domain of medicine, should be sullied by misrepresentation, bitter partisanship, sorrow and suicide.

We shall make no attempt to enter into this fifty-year old debate, but will give only the facts in the case of each claimant. On March 30, 1842, Dr. Crawford W. Long, a respectable practitioner of Jefferson, Ga., excised a tumor on the person of one James Venable, under sulphuric ether anæsthesia.

On July 3, 1842, Dr. Long amputated the toe of a negro boy for disease, using ether. On September 9, 1843, he extirpated a tumor from head of Mary Vincent of Jackson, Ga., and on January 8, 1845, he amputated two fingers of a negro boy. The certificates of two witnesses to this operation state that the first finger was removed under the anæsthetic and that the boy experienced no pain; that the second was removed without ether and was very painful. So much for Long's claims

On December 11, 1844, Horace Wells, a Hartford dentist, had a tooth removed under anæsthesia from nitrous oxide gas administered by by G. Q. Calton. Wells' claim rests upon the uncertain support of a decayed tooth. The claim is now as decayed as was the tooth.

The sole pretense of the third claimant, Dr. Jackson, to priority of discovery, is founded upon the alleged claim of having suggested to Morton to use sulphuric ether in place of nitrous oxide gas. It would appear that Jackson's pretenses secured a hearing more through the efforts of rich and influential friends than by reason of their own merits.

On October 16, 1846, Dr. W. T. G. Morton, of Boston, administered ether to a pâtient at the Massachusetts General Hospital. The patient was about to undergo an operation for removal of a tumor of the neck. The growth was painlessly removed by Dr. J. C. Warren, and the God-given boon of anæsthesia at once became the property of all humanity. The news rapidly ran around the world. Honors and decorations from home and abroad were showered upon Morton. Congress was on the point of voting him a large sum of money. Morton's cupidity was aroused, and he sought to patent ether under the name of "Letheon." At this juncture, Long and Wells and Jackson appeared and pressed their claims with assiduity. The world withheld its judgment, and has not yet rendered the final verdict in favor of any of the contestants.

From the above bare facts, the following conclusions may be legitimately drawn: That the contest lies between Long and Morton; that the claims of Wells and Jackson are insufficiently supported and are almost too gauzy to entitle them to respectful hearing—they have made out no case; that the first to use ether for the relief of the pain of surgical operations was Dr. Long, whose operation in the spring of 1842 was nearly four years before the successful experiment of Morton in October, 1846; and, finally, that Dr. Long is the true discoverer of anæsthesia or painless surgery.

This fact remains as solid as a rock and no amount of opinion or evidence has ever been able to controvert it, or ever shall. That this fact was not universally recognized and acknowledged was Long's own fault. There was, perhaps, some extenuation of it. Living in a remote village, removed from communication and opportunities for the exchange of thought with his brother physicians, without which the life of a practitioner of medicine is one of utter stagnation, and inexpert with the pen, Dr. Long probably did not realize to its fullest the magnitude and far-reaching possibilities of his discovery and did not publish it to the world. Had he had the advantages of residence in a city and the opportunity of an impressive public demonstration, or even had he possessed the native endowments of a mind quick to grasp the strong points of a subject, there would have been no altar erected to the "Unknown God," and the claims of others to the discovery, would have never come to vex the mind and confuse the judgment. But Long let the time go by and fell into the monotonous rut of a country doctor. Then came Morton, who had the alertness and shrewdness to see the value of anæsthesia, and forestalled the procrastinating countryman. Had this not occurred and had not Long been sharply aroused from his lethargy by the laudations of Morton, he would have probably continued on in silence, making no announcement, and the uncrystallized knowledge of the great secret would have remained locked in his breast and have died with him.

Morton thus became the involuntary and unconscious mouthpiece of Long, in announcing to the world that which Long had told and shown to friends four years before.

Between Long and Morton is the difference between the discoverer and elaborator.

Of the two, more credit is due Morton. Long hid his light under a bushel; Morton flashed it from the hill-tops.

*Die wahrheit soll die Losung alle Widerspruche sein!*

